# Experimental Evaluation of the Availability of LoRaWAN Frequency Channels in the Czech Republic

**DOI:** 10.3390/s21030940

**Published:** 2021-01-31

**Authors:** Vojtěch Novák, Michal Stočes, Tereza Čížková, Jan Jarolímek, Eva Kánská

**Affiliations:** Faculty of Economics and Management, Czech University of Life Sciences Prague, Kamycka 129, 165 00 Praha, Czech Republic; stoces@pef.czu.cz (M.S.); cizkovat@pef.czu.cz (T.Č.); jarolimek@pef.czu.cz (J.J.); kanska@pef.czu.cz (E.K.)

**Keywords:** LoRaWAN, LPWAN, IoT, signal, ESRI, GIS, ArcGIS insights, frequency, device, experiment

## Abstract

LoRaWAN communication allows you to create IoT (Internet of Things) solutions across many disciplines. A specific field of application is precision agriculture, which demands this technology mainly due to the fact that it is possible to create low power sensor devices with it. However, in densely populated areas, a lower success rate of message delivery can be observed on some communication channels. For example, this can have an impact on urban agriculture projects. After performing an experiment and analytical–statistical data processing using the Geographic Information System (GIS) tool ArcGIS Insights, it was shown that the success of message delivery on the basic LoRaWAN channel (868.3 MHz) is lower than for the others. Therefore, to ensure higher reliability and thus energy savings, it is appropriate to optimize the use of frequency channels.

## 1. Introduction

LoRaWAN communication can be used for example in areas such as smart cities, industrial applications, precision agriculture, and many more. It is very important for further research to gain detailed experience from the practical deployment of LoRaWAN technology. Based on this knowledge, the technology itself can be improved, or other monitoring solutions can be found.

In the context of my work and activities at the Czech University of Life Sciences within the solved agricultural projects of my colleagues from the university, this work focuses on the evaluation of technological environments for these research projects aimed at the field of agriculture. For example, our university team has previously published that: “IoT technologies are currently the most developing area of telemetry transmissions in both industrial and agricultural environments” [1]. In most cases, it is essential for these projects that the equipment be a so-called low power, such as monitoring beehives, using beehive scales measuring the weight of the beehive and some other parameters—for example, indoor temperature and relative humidity of the hive [2].

The success of these projects often depends on the reliability of the used technologies and also on its appropriate use. Thanks to the availability of LoRaWAN technology in the Czech Republic with the use of a nationwide operator, it is possible to consider using it for the needs of agriculture within the Czech Republic.

LoRaWAN wireless technology offers a Star topology using stochastic network access (Aloha) at the wireless level. Spread spectrum modulation with an optional spreading factor parameter is used. A message sent by one device is received on many different gateways when it is forwarded to a central point of the network, the so-called network server, which combines the individual messages into one [3].

## 2. Related Work

A review of the available literature has shown that many studies and experiments have been published. For example, a significant factor affecting all Low Power Wide Area Networks (LPWAN) is the legislative restrictions addressed for example as Impact of EU duty cycle and transmission power limitations [4]. An alternative technology to LoRaWAN is Sigfox [5], which it compares with each other by Vejlgaard et al. [6].

The importance of the use of LoRaWAN technology in agriculture can be observed, for example, in the study, “A Case Study in Kenya [7] and LoRaWAN in Industrial 4.0 Environments” [8].

Technology is often studied in laboratory conditions, as it is able to function on its own, for example in terms of capacity as stated by many authors, e.g., [6,7]. Martina Capuzzo created a mathematical model describing LoRaWAN Performance with Bidirectional Traffic [9].

For example, practical verification of the spread of LoRaWAN within the university campus can be found in Rabey Anzum, who worked with the 3D ray tracing method [9], similar to the team from the University of Duisburg-Essen [10]. However, most studies focus on a targeted verification of technological possibilities, or on improving Quality-Of-Service in LoRaWAN through Optimized Radio Resource Management as settings parameters (ADR, SF, CSS, channels, etc.) [10,11,12]. The consequences of such optimization have an impact on energy consumption, which is also addressed by several authors, as exemplified: “Comparison of LoRaWAN Classes and their Power Consumption” [13].

To a small extent, the results of operating parameters in the actual deployment of technology outside the laboratory environment are published, such as the work of an Italian team led by Lorenzo Parri, who tested the possibilities of using connectivity at sea [14]. However, interference cannot be expected there, as is the case, for example, in the city center, and this is the topic of this article. This article went a different way, namely to obtain operational metadata from the actual installation of the sensor, where the payload itself is used for another experiment, and find out what information these metadata are able to provide. During the testing of the usability of the LoRaWAN technology for the needs of Urban Agriculture, a three-day measurement was performed in Prague, the capital of the Czech Republic. After analytical and statistical data processing, some interesting phenomena were revealed. This work tries to approach them, but it does not solve their direct cause; instead, it tries to establish a hypothesis.

## 3. Materials and Methods

Metadata obtained from the LoRaWAN network of the Czech operator called České Radiokomunikace in a special test mode were used for the experiment. The device used a Murata communication module [15] with a built-in network layer. Thus, the behavior of the device on the network is given by the manufacturer Murrata. The device sent a data message approximately every two minutes, and 1000 individual received messages were selected for the experiment to obtain data for approximately three days of the experiment. The legislative duty cycle limit was not reached; sending one message took 51 ms and 92 ms, depending on the current data rate. The experiment did not evaluate the success of delivery, but it did conduct a mutual statistical comparison of successfully received messages.

Necessary transformations were performed with the data. The data structure provided by the network is structured into a JSON document with a variable message length. The structure of the messages thus received includes a common data part comprising attributes e.g., Receive timestamp, Data rate used, Communication frequency (channel), Message transmission time, Frame counter, sequence number, etc. For the part with the metadata field obtained from individual gateways, for each gateway, its ID, its geographical location, signal parameters RSSI (Received Signal Strength Indication) and SNR (Signal-to-Noise Ratio), and some other attributes are given. In order to be able to process the data in the intended analytical tool, a new data structure had to be designed. The data were converted to GeoJSON data format using the function in Appendix B, which were further supplemented with the position information of the place where the sensor was located; also, a unique message identifier was created by combining “seqno” and “fcnt” attributes, which was added to the respective GW messages so that the data could be divided into two GeoJSON files.

As this is data containing position information, the Geographic Information System (GIS) platform, specifically ArcGIS Insights, was used to analyze it, into which the files prepared in this way can be loaded. In order to analyze the data together, a prepared identifier was used to create a 1:N session, which corresponds to the fact that a message sent by one device was received by many gateways.

For processing and analyzing the data, we used the ArcGis Insights tool, which allows a direct data connection. This tool offers many easily accessible tools for working with data. In particular, it is possible to visualize data using graphs and maps. Data can be read in MS Excel format or in GeoJSON format, and data can be composed in the tool using sessions. Individual views are created from the prepared datasets using drag-and-drop. Individual graphic elements offer dynamic redrawing based on the selection in the linked element [16].

A very useful tool that was used for the analysis is the Boxplot view. A description of the meaning of individual elements is available on the manufacturer’s website [17].

## 4. Results

### 4.1. Experiment introduction

**Figure A1:**
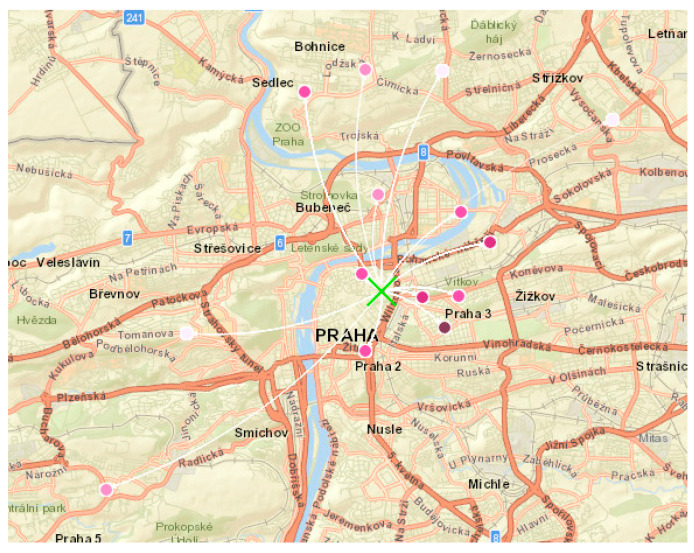
This image shows the geographical location of all 14 gateways that received a message from the device during the experiment. The color range of the dots corresponds to the frequency of received messages. The green cross shows the place from which it was broadcast.

This section describes basic information about the course and duration of the experiment. The experiment lasted for the period from 6:40 p.m. 23 November 2020 to 9:20 a.m. 25 November 2020, where the operator-certified testing device sent a data message every two minutes. The device had ADR activated, and during the experiment, the network server set up automatically the Spreading Factor value, which ranged between SF7 and SF8. The device was located approximately in the city center, and during the entire experiment, messages sent by the device were delivered to 14 gateways of the largest Czech LoRaWAN operator, as shown in Figure A1 in Appendix A.

According to the value of the frame counter contained in the metadata, it is possible to determine the total success rate of message delivery, which reached 84%, where 1192 messages were sent for 1000 successfully received messages. Undelivered messages are distributed evenly, where the maximum number of undelivered consecutive messages was 4, and only in one case. A detailed view of undelivered messages is indicated in the following Table 1 below.

### 4.2. Signal Analysis

**Figure 1:**
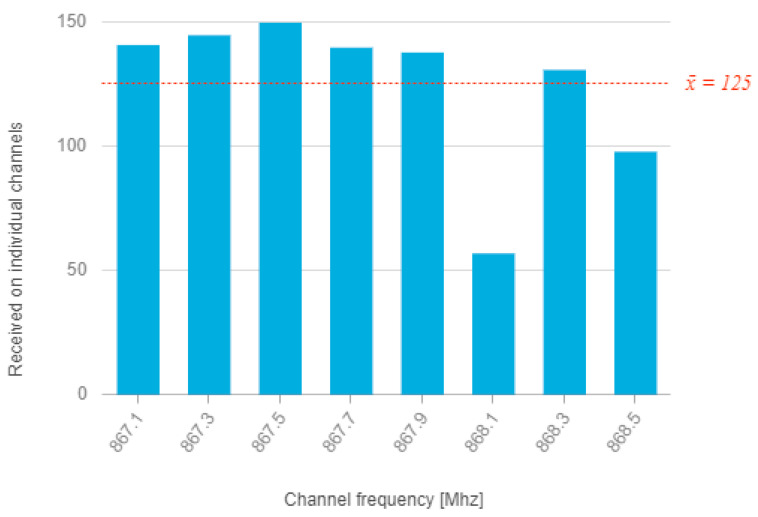
Number of received messages within individual LoRaWAN channels.

**Figure A2:**
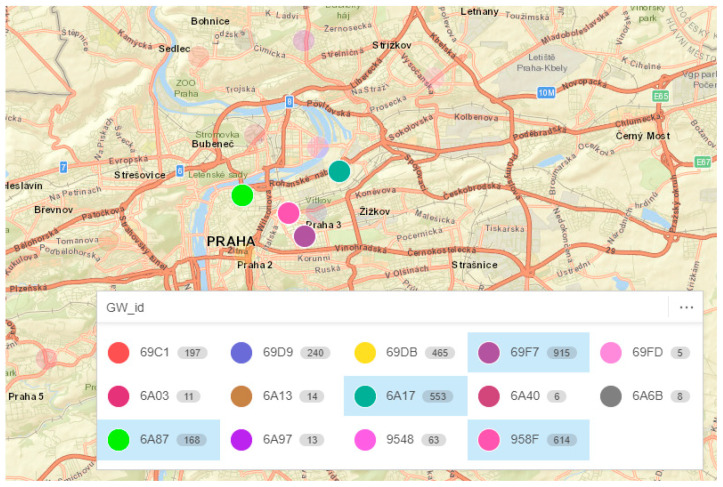
In the picture, you can see the table of individual gateways, where the number in the gray field indicates the number of received messages. The blue-colored fields are those gateways that have received a message on the frequency of 868.1 MHz.

Subsequently, the data were analyzed analytically in ArcGIS Insights, and one of the outputs that was focused on evaluating the success of message delivery showed that one first basic channel (868.1 Mhz) shows a significantly below-average number of received messages. For this channel, the GW was displayed on the map (in Appendix A, Figure A2) that received the message on this channel. An overview is shown in Figure 1.

**Figure 2:**
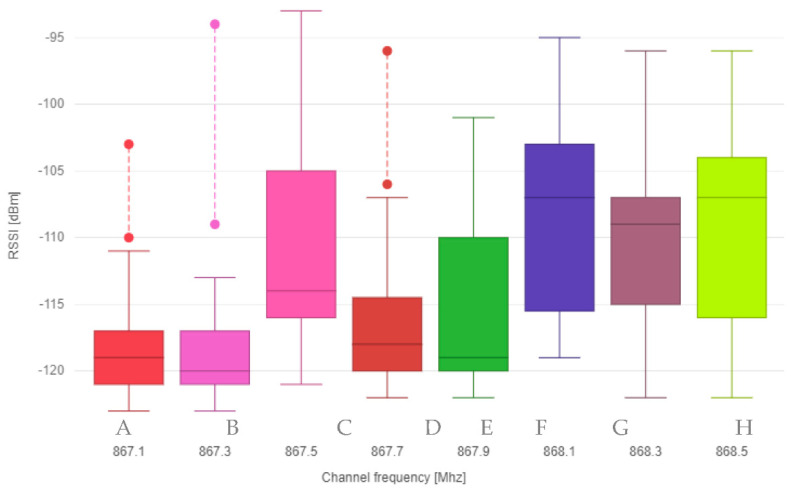
Boxplot graph for frequency channel and RSSI.

**Figure 3:**
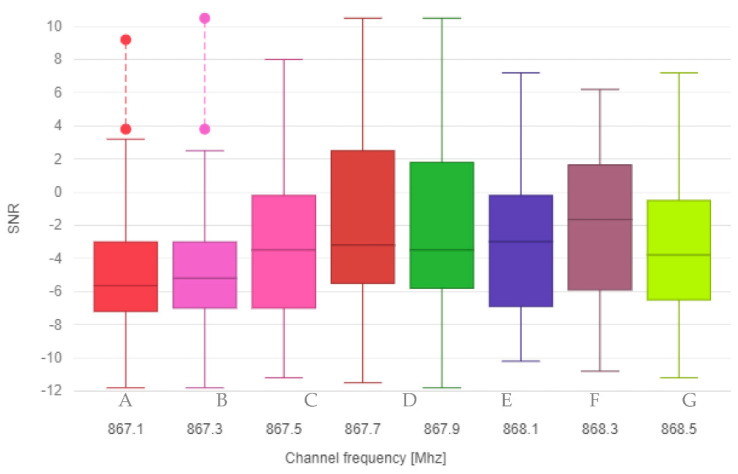
Boxplot graph for frequency channel and SNR.

The data were further statistically processed and visualized using two boxplot graphs, where the first graph shows the relationship between the specific frequency channel and RSSI received messages (Figure 2), and the second graph shows the relationship between frequency channel and SNR (Figure 3).

**Figure 4:**
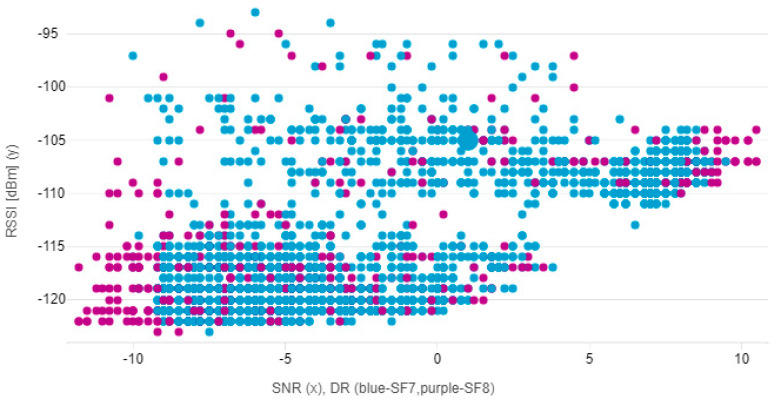
Scatter plot shows the relationship between signal strength and signal-to-noise ratio. The color resolution shows the currently selected spreading factor.

The interference hypothesis is also supported by Figure 4, where the relationship between signal strength and SNR. The spreading factor, was set to automatic and ranged between SF7 and SF8, which is marked in color in the graph.

## 5. Discussion

This study describes the detected state measured in a real environment, which was analytically processed. The analysis revealed anomalies compared to theoretical expectations. Thus, an uneven distribution of received messages in individual channels. The most likely cause of the lower proportion of messages delivered on the 868.1 Mhz and 868.5 MHz channels will be local interference from other traffic in this ISM band. This assumption can be verified by direct measurement using a spectrum analyzer, but such measurement would have to take place simultaneously over a large area.

This result is also interesting to evaluate depending on the LoRaWAN specification, where it is given that channels corresponding to 868.1, 868.3, and 868.5 MHz shall be implemented but cannot be modified, according to the protocol specification [18]. Adding other channels in larger quantities can increase delivery success. Mitigation techniques can also be discussed in the contribution to reliability [19], but the article focuses on the practical life of currently commercially available technology; it is not possible to consider complex methods that are not supported in the standard, but as [20] indicated, improving the reliability could have a significant positive impact on energy consumption.

It is not possible to exclude these channels from use, although it would be possible to increase the success of message delivery. The observed results may also be related to the conclusion of the work of Georgiou and Raza, who investigated the effects of interference in a single gateway LoRa network [21]. Researchers at the University of Tokyo say that by detecting interference, the delivery success rate can be increased by 10% [22]. Frequency interference modeling was also handled by the French team [23], and the issue of mutual interference (here collisions) can also be found in the work “Improving channel utilization of LoRaWAN by using novel channel access mechanism” [24].

Frequency interference modeling is also being investigated by the French team [20], and the issue of mutual interference (collisions) can also be found in the study “Improving channel utilization of LoRaWAN by using a new channel access mechanism” [21], the results of which do not indicate the cause of the significant decrease in success in delivery on basic channels.

## Figures and Tables

**Table 1:** Numbers of consecutive undelivered messages broken down by groups.

Group ^1^	Occurrence	Undelivered
1	136	136
2	23	46
3	2	6
4	1	4

^1^ The group value indicates the number of undelivered messages in a row.

## Data Availability

The data presented in this study are available on request from the corresponding author. The data are not publicly available due to he data contains sensitive data, in particular accurate location information about the third party infrastructure.

## Appendix B

The following code shows the transformation of a message received from a network server into GeoJSON format.

1.  function toGeoJSON(item, index) {
2.    var coordinates_id = item.seqno.toString() + ”-” + item.fcnt.toString()
3.    for (i = 0; i < item.gws.length; i++) {
4.      var dato = {
5.        ”id”: id,
6.        ”geometry”: {
7.          ”type”: ”Point”,
8.          ”coordinates”: [
9.            item.gws[i].lon,
10.            item.gws[i].lat
11.          ],
12.          ”crs”: {
13.            ”type”: ”name”,
14.            ”properties”: {
15.              ”name”: ”EPSG:4326”
16.            }
17.          }
18.        },
19.        ”properties”: {
20.         ”rssi”: item.gws[i].rssi,
21.         ”snr”: item.gws[i].snr,
22.         ”ts”: new Date(item.gws[i].ts).toISOString().replace(‘T’,’’).substr(0, 23),
23.         ”tmms”: item.gws[i].tmms,
24.         ”gweui”: item.gws[i].gweui,
25.         ”signal_index”: item.gws[i].signal_index,
26.         ”coordinates_id”: coordinates_id
27.        },
28.        ”type”: ”Feature”
29.      }
30.      id++
31.      features.push(dato);
32.    }
33.  }
